# Metformin reduces gastric cancer risk in patients with type 2 diabetes mellitus

**DOI:** 10.18632/aging.101019

**Published:** 2016-08-30

**Authors:** Chin-Hsiao Tseng

**Affiliations:** ^1^ Department of Internal Medicine, National Taiwan University College of Medicine, Taipei, Taiwan; ^2^ Division of Endocrinology and Metabolism, Department of Internal Medicine, National Taiwan University Hospital, Taipei, Taiwan; ^3^ Division of Environmental Health and Occupational Medicine of the National Health Research Institutes, Zhunan, Taiwan

**Keywords:** gastric cancer, diabetes mellitus, metformin, Taiwan

## Abstract

This retrospective cohort study investigated whether metformin may reduce gastric cancer risk by using the reimbursement databases of the Taiwan's National Health Insurance. Patients with type 2 diabetes diagnosed during 1999-2005 and newly treated with metformin (*n*=287971, “ever users of metformin”) or other antidiabetic drugs (*n*=16217, “never users of metformin”) were followed until December 31, 2011. The effect of metformin (for ever versus never users, and for tertiles of cumulative duration of therapy) was estimated by Cox regression incorporated with the inverse probability of treatment weighting using propensity score. Results showed that the respective numbers of incident gastric cancer in ever and never users were 759 (0.26%) and 89 (0.55%), with respective incidences of 55.26 and 122.53 per 100,000 person-years. The overall hazard ratio (95% confidence intervals) of 0.448 (0.359-0.558) suggested a significantly lower risk among ever users. In tertile analyses, hazard ratios (95% confidence intervals) for the first (<21.47 months), second (21.47-45.97 months) and third (>45.97 months) tertile of cumulative duration was 0.973 (0.773-1.224), 0.422 (0.331-0.537) and 0.120 (0.090-0.161), respectively, while compared to never users. In conclusion, metformin significantly reduces gastric cancer risk, especially when the cumulative duration is more than approximately 2 years.

## INTRODUCTION

According to the latest statistical data, 951,600 new cases of gastric cancer and 723,100 deaths ascribed to gastric cancer occurred around the world in 2012 [1]. The incidence of gastric cancer is twice as high in men as in women, and the highest incidence occurs in Eastern Asia, Central and Eastern Europe, and South America [1]. Diabetes mellitus is associated with a significantly higher risk of gastric cancer [2-4] and infection with *Helicobacter pylori* (HP) [5]. Chronic infection with HP has been identified as the most important risk factor of gastric cancer [1], and eradication therapy of HP infection reduces the incidence of gastric cancer in most parts of the world [1].

Other risk factors of gastric cancer include salt intake, smoking and obesity [1]. A recent study in China suggested an association with hepatitis B virus (HBV) infection [6]. Additionally, some medications commonly used by patients with type 2 diabetes mellitus (T2DM) may have a favorable effect on gastric cancer, including statin [7], aspirin and/or non-steroidal anti-inflammatory drugs (NSAID) [8] and angiotensin converting enzyme inhibitor/angiotensin receptor blocker (ACEI/ARB) [9]. In contrast to other antidiabetic drugs (including sulfonylurea, insulin, thiazolidinediones and incretin-based therapies) that may show an increased risk of cancer [10-16], metformin was first noted to be associated with a reduced risk of cancer in an observational study in 2005 [17]. Metformin has been shown to inhibit the growth and proliferation of cancer cells including the breast [18], endometrium [19], ovary [20], lung [21], thyroid [22], liver [23], pancreas [24], esophagus [25], stomach [26], colon [25], prostate [27], bladder [28], glioblastoma [29], and leukemic cells [30]. In consistent with findings in animals which showed a beneficial effect of metformin on the inhibition of carcinogenesis in at least 17 target organs [31], epidemiological studies demonstrated a protective effect of metformin on a variety of cancer types including thyroid cancer [32], oral cancer [33], colon cancer [34], breast cancer [35], endometrial cancer [36], ovarian cancer [37], prostate cancer [38], bladder cancer [39], kidney cancer [40] and cervical cancer [41]. However, whether metformin may reduce the risk of gastric cancer has not been extensively studied. A previous retrospective cohort study using the reimbursement databases of the National Health Insurance (NHI) in Taiwan suggested a neutral effect of metformin on gastric cancer, with an adjusted hazard ratio (HR) of 1.41 [95% confidence interval (CI): 0.42-4.73] [42]. On the other hand, a Korean study demonstrated a reduced risk of gastric cancer in patients with T2DM who had been using metformin for >3 years and not being treated with insulin (adjusted HR 0.57, 95% CI: 0.37-0.87) [43]. Another recent Italian study suggested a minor but significant risk reduction associated with metformin use (adjusted HR 0.990, 95% CI: 0.986-0.994) [44].

Therefore, studies on the effect of metformin on gastric cancer risk in humans are still rare and the findings are controversial. By using the reimbursement databases of the NHI in Taiwan, the purpose of the present study was to evaluate whether metformin use in patients with T2DM might reduce the risk of gastric cancer. Ever users of metformin were compared to never users of metformin and dose-response relationship was analyzed by using the tertile cutoffs of cumulative duration of metformin therapy. The most important risk factor of HP infection was considered as one of the potential confounders, and the effects of concomitant use of medications including other oral antidiabetic drugs, insulin, statin, fibrate, aspirin, NSAID, ACEI/ARB and calcium channel blockers were also adjusted for. To solve the potential problem of “prevalent user bias” [45], newly diagnosed diabetes patients and incident users of metformin were recruited. To reduce the potential risk of “immortal time bias” (the initial period of follow-up during which the outcome can not occur) [45], patients who were followed for a short period of time (i.e., <180 days) were excluded. To avoid the potential confounding from the differences in baseline characteristics associated with treatment allocation in non-random observational studies, Cox regression models incorporated with the inverse probability of treatment weighting (IPTW) using propensity score (PS) were created [46]. To evaluate whether the findings could be consistent, sensitivity analyses were also conducted by using traditional Cox regression models, comparing users of metformin as the first antidiabetic drug after diabetes diagnosis (defined as “new users”) to never users, and in subcohorts of metformin users and never users with well-matched baseline characteristics.

## RESULTS

### Baseline characteristics

**Figure 1:**
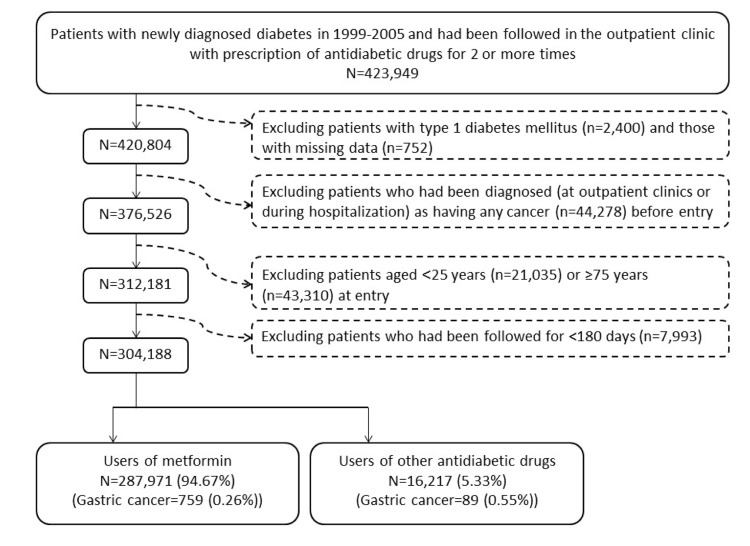
Flowchart showing the procedures in selecting the original sample into the study.

There were 16217 never users and 287971 ever users in the original sample (Figure 1). In the original sample, all baseline characteristics (defined at the time of censor) of the two groups differed significantly, except for hypertension, pioglitazone, Epstein-Barr virus (EBV)-related diagnoses and HBV infection (Table 1). Ever users were characterized by younger age, less males, higher proportions of dyslipidemia, obesity, eye disease, peripheral arterial disease and tobacco abuse, lower proportions of nephropathy, stroke, ischemic heart disease, chronic obstructive pulmonary disease, alcohol-related diagnoses, history of HP infection, and hepatitis C virus (HCV) infection, higher proportions of use of rosiglitazone, ACEI/ARB, statin, fibrate, aspirin and NSAID, but lower proportions of using other antidiabetic medications and calcium channel blocker.

**Table 1:** Comparison of characteristics between metformin never users and ever users in the original sample and in the propensity score matched sample

Variable	Original sample						Matched sample					
	Never users (*n*=16217)		Ever users (*n*=287971)		*P*	SD	Never users (*n*=16217)		Ever users(*n*=16217)		*P*	SD
	*n*	%	*n*	%			*n*	%	*n*	%		
**Demographic data**												
Age (years)*	63.61±10.42		61.39±10.22		<0.0001	−22.03	63.61±10.42		63.98±9.97		0.0011	4.29
Sex (men)	9297	57.33	155122	53.87	<0.0001	−7.28	9297	57.33	9261	57.11	0.6862	−0.76
Occupation												
I	6336	39.07	116153	40.33	<0.0001		6336	39.07	6417	39.57	0.4435	
II	3230	19.92	65960	22.91		7.63	3230	19.92	3197	19.71		−0.55
III	3403	20.98	56159	19.50		−3.71	3403	20.98	3301	20.36		−1.29
IV	3248	20.03	49699	17.26		−7.58	3248	20.03	3302	20.36		0.68
Living region
Taipei	5455	33.64	97260	33.77	<0.0001		5455	33.64	5457	33.65	0.4721	
Northern	1659	10.23	34434	11.96		5.68	1659	10.23	1615	9.96		−0.98
Central	2838	17.50	51314	17.82		0.89	2838	17.50	2853	17.59		0.27
Southern	2805	17.30	46172	16.03		−3.51	2805	17.30	2723	16.79		−1.11
Kao-Ping/Eastern	3460	21.34	58791	20.42		−2.28	3460	21.34	3569	22.01		1.84
**Major comorbidities**												
Hypertension	13307	82.06	234744	81.52	0.0850	−1.46	13307	82.06	13361	82.39	0.4329	1.17
Dyslipidemia	11722	72.28	239064	83.02	<0.0001	27.55	11722	72.28	11681	72.03	0.6115	0.00
Obesity	440	2.71	16493	5.73	<0.0001	15.14	440	2.71	391	2.41	0.0851	−1.84
**Diabetes-related complications**												
Nephropathy	5661	34.91	77162	26.80	<0.0001	−19.03	5661	34.91	5561	34.29	0.2431	−2.08
Eye disease	3007	18.54	89012	30.91	<0.0001	29.37	3007	18.54	2696	16.62	<0.0001	−5.28
Stroke	5403	33.32	83303	28.93	<0.0001	−10.04	5403	33.32	5371	33.12	0.7060	−0.45
Ischemic heart disease	7771	47.92	130742	45.40	<0.0001	−5.32	7771	47.92	7835	48.31	0.4769	0.94
Peripheral arterial disease	3774	23.27	72114	25.04	<0.0001	4.28	3774	23.27	3744	23.09	0.6930	−0.67
**Antidiabetic drugs**												
Insulin	1351	8.33	6097	2.12	<0.0001	−29.93	1351	8.33	968	5.97	<0.0001	−10.41
Sulfonylurea	11790	72.70	189763	65.90	<0.0001	−11.38	11790	72.70	12153	74.94	<0.0001	7.29
Meglitinide	1337	8.24	10346	3.59	<0.0001	−20.88	1337	8.24	1313	8.10	0.6266	−0.30
Acarbose	1833	11.30	14527	5.04	<0.0001	−22.51	1833	11.30	1758	10.84	0.1844	−1.52
Rosiglitazone	480	2.96	12954	4.50	<0.0001	8.58	480	2.96	473	2.92	0.8180	−0.13
Pioglitazone	401	2.47	7014	2.44	0.7659	0.46	401	2.47	434	2.68	0.2473	1.36
**Potential risk factors of gastric cancer**												
COPD	8087	49.87	140537	48.80	0.0083	−2.53	8087	49.87	8180	50.44	0.3017	1.22
Tobacco abuse	460	2.84	11333	3.94	<0.0001	6.26	460	2.84	417	2.57	0.1410	−1.72
Alcohol-related diagnoses	1284	7.92	20186	7.01	<0.0001	−4.23	1284	7.92	1173	7.23	0.0198	−3.09
History of HP infection	5459	33.66	86521	30.05	<0.0001	−8.58	5459	33.66	5478	33.78	0.8234	−0.11
EBV-related diagnoses	116	0.72	2057	0.71	0.9884	−0.05	116	0.72	109	0.67	0.6396	−0.55
HBV infection	730	4.50	12068	4.19	0.0551	−1.87	730	4.50	698	4.30	0.3864	−1.13
HCV infection	1056	6.51	14723	5.11	<0.0001	−6.51	1056	6.51	1018	6.28	0.3884	−1.05
**Medications that are commonly used in diabetes patients and may affect cancer risk**												
ACEI/ARB	11292	69.63	209213	72.65	<0.0001	6.85	11292	69.63	11330	69.86	0.6460	0.65
Calcium channel blocker	10215	62.99	170487	59.20	<0.0001	−8.04	10215	62.99	10232	63.09	0.8450	0.34
Statin	8767	54.06	189092	65.66	<0.0001	24.94	8767	54.06	8642	53.29	0.1639	−1.15
Fibrate	5547	34.20	122858	42.66	<0.0001	18.20	5547	34.20	5419	33.42	0.1330	−1.37
Aspirin	9332	57.54	175607	60.98	<0.0001	7.14	9332	57.54	9241	56.98	0.3071	−0.88
NSAID	16198	99.88	287787	99.94	0.0106	1.76	16198	99.88	16201	99.90	0.6119	0.63

* Age is expressed as mean ± standard deviation

Refer to “Materials and Methods” for the classification of occupation SD: standardized difference, COPD: chronic obstructive pulmonary disease, HP: Helicobacter pylori, EBV: Epstein-Barr virus, HBV: hepatitis B virus, HCV: hepatitis C virus, ACEI/ARB: angiotensin converting enzyme inhibitor/angiotensin receptor blocker, NSAID: non-steroidal anti-inflammatory drugs (excluding aspirin)

It is evident that the baseline characteristics between never users and ever users of metformin were more comparable in the matched sample. Only 5 variables remained significantly different between the two groups, i.e., age, eye disease, insulin, sulfonylurea, and alcohol-related diagnoses. While examining the standardized differences, 12 out of the 31 variables had values >10% in the original sample, but only insulin had a value >10% in the matched sample. These findings suggested that results derived from the matched sample would be less likely influenced by residual confounding from the differences in the baseline characteristics.

### Incidences of gastric cancer and hazard ratios by metformin exposure

Table 2 shows the incidences of gastric cancer by metformin exposure and the hazard ratios comparing metformin exposed to unexposed patients in the original sample and the matched sample. Users of metformin were defined either as ever users or new users (i.e., the first antidiabetic drug was metformin after diabetes diagnosis), and hazard ratios were estimated by IPTW and traditional Cox models. While defined as ever users, the respective number of incident gastric cancer for ever users and never users in the original sample was 759 and 89, with respective incidence of 55.26 and 122.53 per 100,000 person-years. When evaluating the distribution of the incident cases of gastric cancer by the tertiles of cumulative duration of metformin therapy, there was a trend of decreasing incidence with longer duration of exposure. The overall HR showed a significantly lower risk of gastric cancer associated with metformin use in both the IPTW models and the traditional Cox models in either the original sample or the matched sample. When analyzed by the tertiles of cumulative duration of metformin therapy, although a significantly increased risk could be observed for the first tertile, a reduced risk was observed for the second and third tertiles in all models.

**Table 2:** Incidences of gastric cancer and hazard ratios by metformin exposure defined as ever users and new users in the original sample and the matched sample, respectively

Metformin use	*n*	*N*	Person-years	Incidence rate(per 100,000 person-years)	IPTW model			Traditional Cox model		
					HR	95% CI	*P* value	HR	95% CI	*P* value
**I. Metformin defined as ever users**											
1. Original sample											
Never users	89	16217	72632.75	122.53	1.000			1.000		
Ever users	759	287971	1373391.78	55.26	0.448	(0.359-0.558)	<0.0001	0.577	(0.460-0.724)	<0.0001
**Tertiles of cumulative duration of metformin therapy (months)**											
Never users	89	16217	72632.75	122.53	1.000			1.000		
**<**21.47	418	95238	344656.90	121.28	0.973	(0.773-1.224)	0.8147	1.351	(1.068-1.710)	0.0121
21.47-45.97	250	94862	472376.42	52.92	0.422	(0.331-0.537)	<0.0001	0.548	(0.428-0.702)	<0.0001
**>**45.97	91	97871	556358.46	16.36	0.120	(0.090-0.161)	<0.0001	0.161	(0.120-0.217)	<0.0001
2. Matched sample											
Never users	89	16217	72632.75	122.53	1.000			1.000		
Ever users	63	16217	76772.60	82.06	0.668	(0.484-0.923)	0.0145	0.650	(0.470-0.898)	0.0089
**Tertiles of cumulative duration of metformin therapy (months)**											
Never users	89	16217	72632.75	122.53	1.000			1.000		
**<**20.93	37	5349	18972.30	195.02	1.584	(1.078-2.329)	0.0193	1.565	(1.061-2.310)	0.0239
20.93-45.83	19	5354	26489.15	71.73	0.583	(0.355-0.956)	0.0326	0.569	(0.346-0.934)	0.0257
**>**45.83	7	5514	31311.15	22.36	0.179	(0.083-0.386)	<0.0001	0.173	(0.080-0.374)	<0.0001
**II. Metformin defined as new users**											
1. Original sample											
Never users	89	16217	72632.75	122.53	1.000			1.000		
New users	422	153410	724026.21	58.29	0.474	(0.377-0.595)	<0.0001	0.595	(0.469-0.755)	<0.0001
**Tertiles of cumulative duration of metformin therapy (months)**											
Never users	89	16217	72632.75	122.53	1.000			1.000		
**<**21.60	234	50595	180471.52	129.66	1.043	(0.815-1.333)	0.7396	1.373	(1.066-1.768)	0.0142
21.60-45.73	136	50506	248052.67	54.83	0.441	(0.337-0.576)	<0.0001	0.553	(0.420-0.729)	<0.0001
**>**45.73	52	52309	295502.01	17.60	0.134	(0.095-0.189)	<0.0001	0.171	(0.120-0.242)	<0.0001
2. Matched sample											
Never users	89	16217	72632.75	122.53	1.000			1.000		
New users	42	16217	75207.66	55.85	0.455	(0.315-0.657)	<0.0001	0.448	(0.310-0.647)	<0.0001
**Tertiles of cumulative duration of metformin therapy (months)**											
Never users	89	16217	72632.75	122.53	1.000			1.000		
**<**20.07	25	5360	18558.72	134.71	1.087	(0.696-1.699)	0.7126	1.093	(0.697-1.713)	0.6991
20.07-44.67	16	5344	25634.64	62.42	0.507	(0.298-0.864)	0.0125	0.491	(0.288-0.838)	0.0091
**>**44.67	1	5513	31014.30	3.22	0.026	(0.004-0.188)	0.0003	0.026	(0.004-0.186)	0.0003

IPTW: Cox regression incorporated with the inverse probability of treatment weighting using propensity score

HR: hazard ratio, CI: confidence interval

In sensitivity analyses by using metformin new users as the exposure group, the findings were similar to those observed when metformin exposure was defined by ever users.

### Joint effects of metformin and other drugs and HP infection

Table 3 shows the HR for gastric cancer comparing different subgroups of metformin exposure with regards to the exposure of other antidiabetic drugs, statin or HP infection to a referent group who were dual non-users of metformin and another drug or metformin never users and without HP infection. The findings suggested that in the absence of metformin use, the use of the other antidiabetic drugs (Model I to Model VI) or statin (Model VII) did not significantly affect the risk of gastric cancer. However, the risk of gastric cancer was significantly reduced in patients who had been treated with metformin, disregarding the use of other drugs in most of the models. Significant *P* values were observed for the interaction between metformin and sulfonylurea (Model II), acarbose (Model IV), pioglitazone (Model VI) and statin (Model VII). In the model that evaluated the joint effect of metformin and HP infection (Model VIII), it was noted that HP infection significantly increased the risk of gastric cancer disregarding the use of metformin, but the magnitude of the HR associated with HP infection in metformin ever users was much smaller than that in metformin never users. The interaction between metformin and HP infection was significant.

**Table 3:** Models evaluating the potential risk modification on the link between metformin and gastric cancer by other antidiabetic drugs, statin and HP infection

Model	*n*	N	Person-years	Incidence rate(per 100,000 person-years)	HR	95% CI	*P* value
**Model I. Metformin and insulin**							
Metformin (−) / Insulin (−)	74	13307	60371.01	122.58	1.000		
Metformin (−) / Insulin (+)	15	2910	12261.73	122.33	0.884	(0.504-1.549)	0.6667
Metformin (+) / Insulin (−)	558	212749	1001962.79	55.69	0.593	(0.464-0.758)	<0.0001
Metformin (+) / Insulin (+)	201	75222	371428.98	54.12	0.603	(0.456-0.797)	0.0004
						*P*-interaction	0.6877
**Model II. Metformin and sulfonylurea**						
Metformin (−) / Sulfonylurea (−)	16	2416	9478.70	168.80	1.000		
Metformin (−) / Sulfonylurea (+)	73	13801	63154.04	115.59	0.638	(0.370-1.100)	0.1060
Metformin (+) / Sulfonylurea (−)	52	20282	81784.45	63.58	0.403	(0.229-0.709)	0.0016
Metformin (+) / Sulfonylurea (+)	707	267689	1291607.33	54.74	0.416	(0.253-0.686)	0.0006
						*P*-interaction	0.0047
**Model III. Metformin and meglitinide**						
Metformin (−) / Meglitinide (−)	76	13239	59376.40	128.00	1.000		
Metformin (−) / Meglitinide (+)	13	2978	13256.35	98.07	0.734	(0.406-1.328)	0.3067
Metformin (+) / Meglitinide (−)	576	218709	1028883.70	55.98	0.575	(0.451-0.733)	<0.0001
Metformin (+) / Meglitinide (+)	183	69262	344508.08	53.12	0.589	(0.446-0.778)	0.0002
						*P*-interaction	0.7951
**Model IV. Metformin and acarbose**						
Metformin (−) / Acarbose (−)	70	12574	55999.22	125.00	1.000		
Metformin (−) / Acarbose (+)	19	3643	16633.53	114.23	0.907	(0.545-1.510)	0.7076
Metformin (+) / Acarbose (−)	540	181564	838431.60	64.41	0.641	(0.499-0.825)	0.0005
Metformin (+) / Acarbose (+)	219	106407	534960.18	40.94	0.444	(0.336-0.588)	<0.0001
						*P*-interaction	<0.0001
**Model V. Metformin and rosiglitazone**						
Metformin (−) / Rosiglitazone (−)	79	15073	66954.95	117.99	1.000		
Metformin (−) / Rosiglitazone (+)	10	1144	5677.79	176.12	1.726	(0.892-3.340)	0.1054
Metformin (+) / Rosiglitazone (−)	599	226419	1054531.60	56.80	0.631	(0.498-0.801)	0.0001
Metformin (+) / Rosiglitazone (+)	160	61552	318860.18	50.18	0.757	(0.568-1.007)	0.0560
						*P*-interaction	0.1437
**Model VI. Metformin and pioglitazone**						
Metformin (−) / Pioglitazone (−)	83	14651	65029.88	127.63	1.000		
Metformin (−) / Pioglitazone (+)	6	1566	7602.87	78.92	0.638	(0.278-1.464)	0.2888
Metformin (+) / Pioglitazone (−)	603	200948	923382.14	65.30	0.611	(0.484-0.770)	<0.0001
Metformin (+) / Pioglitazone (+)	156	87023	450009.64	34.67	0.367	(0.277-0.487)	<0.0001
						*P*-interaction	<0.0001
**Model VII. Metformin and statin**						
Metformin (−) / Statin (−)	41	7450	32530.71	126.03	1.000		
Metformin (−) / Statin (+)	48	8767	40102.03	119.69	1.092	(0.713-1.673)	0.6860
Metformin (+) / Statin (−)	345	98879	453948.78	76.00	0.758	(0.547-1.051)	0.0971
Metformin (+) / Statin (+)	414	189092	919443.00	45.03	0.530	(0.378-0.744)	0.0002
						*P*-interaction	<0.0001
**Model VIII. Metformin and HP infection**							
Metformin (−)/HP infection (−)	29	10758	49003.82	59.18	1.000		
Metformin (−)/HP infection (+)	60	5459	23628.93	253.93	4.402	(2.819-6.872)	<0.0001
Metformin (+)/HP infection (−)	304	201450	963521.69	31.55	0.708	(0.482-1.039)	0.0779
Metformin (+)/HP infection (+)	455	86521	409870.09	111.01	2.465	(1.685-3.604)	<0.0001
						*P*-interaction	<0.0001

n: case number of incident gastric cancer, N: case number followed HP: Helicobacter pylori, HR: hazard ratio, CI: confidence interval

## DISCUSSION

The findings strongly suggested that metformin significantly reduced the risk of gastric cancer, disregarding the presence or absence of HP infection and independent of other antidiabetic drugs (Tables 2 and 3). The findings are consistent in different analyses and by including only new users of metformin (Table 2).

*n*: case number of incident gastric cancer, *N*: case number followed

HP: *Helicobacter pylori*, HR: hazard ratio, CI: confidence interval

Additionally, a dose-response relationship could well be demonstrated in both the original sample and the matched sample (Table 2).

In the recent Korean study by Kim et al. which retrospectively analyzed the national insurance claims data of 39989 patients with T2DM, metformin use for >3 years was associated with a significant 43% reduction of gastric cancer risk among those who did not use insulin [43]. However, in the present study, it was well demonstrated that the risk reduction associated with metformin use was independent of insulin or other antidiabetic drugs (Tables 2 and 3). It is worthy to note that in the Korean study, gastric cancer risk might be doubled in insulin users while compared to nonuser, disregarding metformin use [43]. Our previous studies may provide some insights for the explanation of the association between insulin use and gastric cancer risk observed in the Korean study. Insulin use was associated with a higher rate of receiving HP eradication therapy, indicating the requirement of insulin for the control of hyperglycemia which could be deteriorated by HP infection (a real risk factor of gastric cancer) [5]. Because the Korean study did not consider the potential confounding of HP infection which might be closely related to insulin use, the higher risk of gastric cancer among insulin users could be explained by a deteriorating hyperglycemia associated with HP infection. Actually, HP infection significantly increased the risk of gastric cancer (Model VIII, Table 3) but insulin did not much affect the risk of gastric cancer (Model I, Table 3) in the present study. The lack of an increased risk of gastric cancer associated with insulin use observed in the present study was also supported by an earlier study conducted in the Taiwanese patients with T2DM [3].

The neutral effect of metformin on gastric cancer risk found in a previous study conducted in Taiwan by using the NHI database could probably be due to the small number of cases included in the study (metformin nonusers: *n*=4327, metformin users: *n*=11390), and the small numbers of incident cases of gastric cancer in metformin users (*n*=24) and users of comparators (*n*=10) [42]. This earlier study also had limitations including a lack of consideration of the potential confounding of HP infection and the incapability to allow an analysis of a dose-response effect because of the small case numbers.

In the Italian study that included a larger sample size of 109255 patients with T2DM, a significant but minor risk reduction of gastric cancer was observed among metformin users (adjusted HR 0.990, 95% CI: 0.986-0.994) [44]. However, this Italian study neither evaluated a dose-response relationship nor considered the potential confounding of HP infection.

It is interesting to observe an increased risk in the first tertiles of cumulative duration of metformin therapy in some of the analyses (Table 2). There are several possible explanations. First, although the important risk factors of obesity and HP infection have both been considered as potential confounders (Table 2), residual confounding from these risk factors could not be completely excluded because we did not have anthropometric data to define obesity and only HP eradication therapy could be used as a surrogate of HP infection. Second, even though the covariates were well matched between ever users and never users of metformin in the matched sample (Table 1), this did not necessarily assure that the distributions of some important risk factors between the first tertile and the referent group would be completely well matched. Therefore, some residual confounding could not be excluded. For example, metformin is always considered as the first-line treatment for patients with T2DM, especially in those with obesity. Patients categorized in the first tertile were short-term users. The increased risk of gastric cancer associated with obesity in patients who were previously on diet control or treated with other medications might be carried over to these short-term users. Third, a recent study interestingly showed that patients with T2DM and HP infection might have more gastrointestinal side effects after taking metformin [47]. Therefore, the duration of metformin therapy might have been shortened if the patients developed HP infection during the course of metformin use.

The mechanisms for a reduced risk of gastric cancer associated with metformin use remains to be explored. Through the activation of 5′-adenosine monophosphate-activated protein kinase (AMPK), metformin inhibits the expression of mammalian target of rapamycin (mTOR), which in turn prevents cell aging and cancer development [48-50]. Metformin has been shown to inhibit cancer cell proliferation in cell cultures [18-30], inhibit carcinogenesis in various strains of rodents [31], reduce the risk of cancer in patients with diabetes [32-41], potentiate the effect of chemotherapeutic agents [51] and improve the survival of patients with cancer [52]. Metformin may also specifically inhibit gastric cancer cell proliferation in both *in vitro* and *in vivo* studies [53]. It inhibits the proliferation of gastric cancer cell lines by blocking cell cycle through the inhibition of cyclins [53], by increasing the expression of phospho-acetyl-CoA carboxylase protein [54], and by inhibiting a gastric cancer-related gene hepatocyte nuclear factor-4α [55]. Metformin may also induce apoptosis in human gastric cancer cells via the inhibition of survivin mediated by mTOR through the activation of AMPK [56] or via the inhibition of hypoxia inducible factor 1α and pyruvate kinase M2 signaling pathway [57]. Metformin may exert a gastric mucosal protection effect [58] and significantly improve gastric ulcer healing mediated by an activation of AMPK [59].

Additionally, the antitumor effect of metformin may also be mediated by increasing the number of CD8^+^ tumor-infiltrating lymphocytes [60] or by impairing one-carbon metabolism acting like an antifolate drug [61]. Gastrointestinal microbiome can influence gastric pathogenesis associated with HP infection by modulating inflammation via the creation of reactive oxygen and nitrogen species [62]. Metformin has recently been shown to induce changes in the com-position of gut microbiome with increased proportion of *Akkermansia muciniphila*, which has an effect on mucin production, restoration of regulatory T cells, down-regulation of IL-1β and IL-6 and improved metabolic profile [63]. Such compositional change in the gut microbiome may modulate the development of intestinal tumor in mice [64]. Whether this change in the gut microbiome may affect the risk of gastric cancer associated with metformin use is an interesting issue of clinical importance awaiting further investigation.

The strengths associated with the use of the nationwide databases of the NHI have been discussed previously [40]. There are some limitations of the study that require discussion here. First, salt intake and obesity can be risk factors of gastric cancer [1] and body mass index is closely associated with cancer mortality [65]. However, we did not have data of anthropometric factors and salt intake for analyses. Second, smoking is also a possible risk factor [1], but we could only use diagnoses of chronic obstructive pulmonary disease and tobacco abuse as surrogates. Third, environmental factors and genetic disposition are all implicated in cancer development, but we could not evaluate the interplay between family history, lifestyle, diet, and genetic parameters. Fourth, we did not have biochemical data to evaluate their impact. Fifth, we did not have information on the pathology, grading and staging of gastric cancer. However, because adenocarcinoma accounts for nearly 90% of all cases of gastric cancer in Taiwan [66], the findings of the present study should better be applied to adenocarcinoma.

In summary, this study is probably the first to clearly show that metformin use among Taiwanese patients with T2DM may significantly reduce the risk of gastric cancer, especially when it has been used for approximately 2 years. The risk reduction associated with metformin shows a dose-response relationship and is not affected by the use of other medications or by HP infection.

## MATERIALS AND METHODS

### NHI reimbursement databases

The NHI is a compulsory and universal system of health insurance implemented in Taiwan since March 1995. The NHI covers >99% of Taiwan's residents and has contracts with >98% of the hospitals nationwide. Computerized and standard claim documents must be submitted to the Bureau of NHI for reimbursement by the contracted medical institutes.

The NHI reimbursement databases have been handled by the National Health Research Institutes (NHRI) and can be used for academic researches if approved by an ethical review board and the NHRI. Individual identification information was scrambled for the protection of privacy. The databases contain detailed records of every visit of each patient (including outpatient visits, emergency department visits and hospital admission) and include principal and secondary diagnostic codes, prescription orders, and claimed expenses.

### Selection of study samples

Figure 1 shows the procedures in recruiting a cohort of patients with newly diagnosed T2DM at an onset age of 25-74 years during the period from 1999 to 2005 into the study (original sample). To assure that diabetes was first diagnosed after 1999, patients who had a diagnosis of diabetes mellitus during 1996-1998 were excluded. Patients should have been followed in the outpatient clinic with prescription of antidiabetic drugs for 2 or more times (*n*=423949). In Taiwan, patients with type 1 diabetes can be issued a so-called “Severe Morbidity Card” after a certified diagnosis and they are waived of much of the co-payment. Patients who held a Severe Morbidity Card certifying they had type 1 diabetes were also excluded (*n*=2400). A total of 752 patients were excluded because of missing data. Patients who had been diagnosed as having any cancer before entry were excluded (*n*=44278). Patients aged **<**25 (*n*=21035) or (**)75 (*n*=43310) years were not included. Patients who had been followed up for **<**180 days (*n*=7993) were also excluded.

In consideration that the baseline characteristics might be imbalanced between metformin ever users and never users in the original sample, a 1:1 matched-pair sample was created based on 8 digits of PS according to the methods described by Parsons (matched sample) [67].

### Definitions of variables

Diabetes was coded 250.XX and gastric cancer 151, based on the *International Classification of Diseases, Ninth Revision, Clinical Modification* (ICD-9-CM). Cumulative duration (months) of metformin use was calculated and tertiles of cumulative duration were used for evaluation of a dose-response effect.

Covariates were defined at the time of censor. Demographic data of age, sex, occupation and living region, and factors that might be correlated with metformin use, diabetes severity or cancer risk were considered as potential confounders. The living region and occupation were classified as detailed elsewhere [34]. In brief, the living region was classified as Taipei, Northern, Central, Southern, and Kao-Ping/Eastern. Occupation was classified as class I (civil servants, teachers, employees of governmental or private businesses, professionals and technicians), class II (people without a specific employer, self-employed people or seamen), class III (farmers or fishermen) and class IV (low-income families supported by social welfare, or veterans). Other potential confounders included [68-70] 1) major comorbidities associated with diabetes mellitus: hypertension (ICD-9-CM code: 401-405), dyslipidemia (272.0-272.4) and obesity (278); 2) diabetes-related complications: nephropathy (580-589), eye disease (250.5, 362.0, 369, 366.41 and 365.44), stroke (430-438), ischemic heart disease (410-414), and peripheral arterial disease (250.7, 785.4, 443.81 and 440-448); 3) antidiabetic drugs: insulin, sulfonylurea, meglitinide, acarbose, rosiglitazone and pioglitazone; 4) potential risk factors of gastric cancer: chronic obstructive pulmonary disease (a surrogate for smoking; 490-496), tobacco abuse (305.1, 649.0 and 989.84), alcohol-related diagnoses (291, 303, 535.3, 571.0-571.3 and 980.0), history of HP infection (defined below), diagnoses related to EBV infection (075, 710.3 and 710.4), HBV infection (070.22, 070.23, 070.32, 070.33 and V02.61) and HCV infection (070.41, 070.44, 070.51, 070.54 and V02.62); and 5) medications that are commonly used in diabetes patients and may potentially affect cancer risk: ACEI/ARB [9], calcium channel blocker [71], statin [7], fibrate [72], aspirin and NSAID (excluding aspirin) [8]. History of HP infection was defined in patients who met one of the following two criteria: 1) patients receiving an HP eradication therapy (detailed previously [5], defined in brief as a combination use of proton pump inhibitor or H2 receptor blockers, plus clarithromycin, metronidazole or levofloxacin, plus amoxicillin or tetracycline, with or without bismuth, in the same prescription order for 7-14 days); and/or 2) HP infection diagnosis (041.86). The accuracy of disease diagnoses in the NHI database has been studied previously. Agreements between claim data and medical records are moderate to substantial, with Kappa values ranged from 0.55 to 0.86 [73].

### Calculation of gastric cancer incidence

The incidence density of gastric cancer was calculated for never users and ever users and for different subgroups of exposure to metformin. The numerator of the incidence was the number of patients with incident gastric cancer during follow-up, and the denominator was the person-years of follow-up. Follow-up started on the first day of the use of antidiabetic drugs and ended on December 31, 2011, at the time of a new diagnosis of gastric cancer, or on the date of death or the last reimbursement record.

### Statistical analyses

Baseline characteristics between never users and ever users were compared by Student's t test for age and by Chi-square test for other variables in the original sample and the matched sample, respectively. Standardized differences for all covariates were calculated using the methods described by Austin and Stuart [74]. A value of standardized difference >10% might indicate meaningful imbalance with potential confounding [74].

Logistic regression was used to create PS from the baseline characteristics shown in Table 1. The treatment effect was estimated by Cox regression incorporated with IPTW using PS [46], in the original sample and the matched sample, respectively. Hazard ratios were estimated for ever users versus never users, and for each tertile of cumulative duration of metformin therapy compared to never users as referent. As sensitivity analyses, traditional Cox regression models were created by setting an entry date on January 1, 2006, and followed patients without gastric cancer diagnosed before this date for 6 years until December 31, 2011.

Metformin may be used as the first antidiabetic treatment after diabetes is diagnosed (new users) or prescribed at any time during the treatment course of diabetes (ever users). In consideration that the findings derived from new users might not be comparable to ever users, sensitivity analyses on the calculation of incidence of gastric cancer and hazard ratios were also conducted by comparing only new users of metformin to never users.

To further examine whether the use of other drugs (i.e, insulin, sulfonylurea, meglitinide, acarbose, rosiglita-zone, pioglitazone and statin, respectively) or HP infection might exert an impact on the association between metformin use and gastric cancer risk, additional analyses were conducted by categorizing metformin ever users into 4 different subgroups: 1) dual non-users of metformin and another drug or metformin never users and without HP infection (as referent groups); 2) metformin (−)/other drug (+) or metformin (−)/HP infection (+); 3) metformin (+)/other drug (−) or metformin (+)/HP infection (−); and 4) metformin (+)/other drug (+) or metformin (+)/HP infection (+). The interactions between metformin use and other medications or HP infection were also tested by estimate-ing the *P* values of their product terms in modeling.

Analyses were conducted using SAS statistical software, version 9.3 (SAS Institute, Cary, NC). *P*<0.05 was considered statistically significant.
